# Evaluation of Xpert Carba-R for detecting carbapenemase-producing organisms in South Africa

**DOI:** 10.4102/ajlm.v12i1.1898

**Published:** 2023-01-05

**Authors:** Sanelisiwe T. Duze, Teena Thomas, Tshegofatso Pelego, Sabelle Jallow, Olga Perovic, Adriano Duse

**Affiliations:** 1 Department of Clinical Microbiology and Infectious Diseases, Faculty of Health Sciences, School of Pathology, University of the Witwatersrand, Johannesburg, South Africa; 2 Infection Control Services Laboratory, National Health Laboratory Services, Charlotte Maxeke Academic Hospital, Johannesburg, South Africa; 3 Centre for Healthcare-Associated Infections, Antimicrobial Resistance and Mycoses, National Institute for Communicable Diseases, Johannesburg, South Africa

**Keywords:** *Enterobacterales*, carbapenemase-producing *Enterobacterales*, carbapenemase, Xpert Carba-R, rectal screening

## Abstract

This study evaluated the performance of the Xpert Carba-R assay for detecting the five common carbapenemases in carbapenemase-producing organisms in Johannesburg, South Africa between April 2021 and September 2021. The assay demonstrated 98% sensitivity and 97% specificity. It was also able to detect all the carbapenemases in double carbapenemase producers, as well as carbapenemases in non-fermenter organisms. The Xpert Carba-R assay, therefore, allows the rapid (< 1 h) and accurate identification of the common carbapenemases in pure bacterial cultures and rectal swabs. This assay can aid in the timeous institution of appropriate treatment and infection prevention and control measures.

## Introduction

The rapid spread of carbapenemase-producing *Enterobacterales* (CPE) is a major threat to public health worldwide.^1^ Moreover, carbapenemases in *Enterobacterales* reside on mobile genetic elements that facilitate their widespread dissemination to other bacterial species or genera, which can spread from person to person, particularly in hospital settings.^2,3^

Carbapenemases have been classified into four classes (A, B, C, and D).^4^ Within these classes, the most common carbapenemases are *Klebsiella pneumoniae* carbapenemases (KPC) in class A, imipenemases (IMP), Verona integrated metallo-β-lactamases (VIM) and New Delhi metallo-β-lactamases (NDM) in class B, and the class D oxacilinases (OXA)-type enzymes (e.g. OXA-48 and its variants).^2,4^

In South Africa, the most dominant carbapenemases are NDM and OXA-48 and its variants.^5,6,7^ A recent report by Perovic et al. revealed an alarming increase in the prevalence of OXA-48 and its variants.^7^ This is concerning as OXA-48 and its variants can be missed by routine antimicrobial susceptibility testing as these isolates demonstrate low-level resistance to the carbapenems.^8^ Therefore, rapid and accurate detection of carbapenemases through rectal screening of high-risk patients is crucial for infection prevention and control measures.^3^ Automated molecular assays such as the Xpert Carba-R (Cepheid, Sunnyvale, California, United States) offer rapid detection of the five common carbapenemases.^9^

To our knowledge, no data has been published on the performance of the Xpert Carba-R assay in the African setting. To adopt this assay in our laboratories, local verification taking into account the local epidemiology and the prevalence of the disease is essential.^10^ Therefore, this study aimed to evaluate the performance of the Xpert Carba-R assay on carbapenemase-producing organisms using cultured isolates and spiked rectal swab specimens in Johannesburg, South Africa.

## Methods

### Ethical considerations

Ethics clearance for this study was obtained from the University of Witwatersrand Ethics Committee (study approval number: M210752). Researchers involved in this study provided stool samples voluntarily, with verbal consent for which written documentation was kept. The stool samples were used as matrices for the preparation of spiked rectal swabs. To maintain confidentiality as well as protect researchers’ privacy, all data were anonymised and no personal identifiers were used.

### Sample collection

The study was conducted at the Infection Control Laboratory Services in Johannesburg, South Africa, between April 2021 and September 2021 using a collection of 95 previously characterised *Enterobacterales* isolates. These included 66 CPE positive for either the *bla*_KPC_, *bla*_NDM_, *bla*_VIM_, *bla*_OXA-48_, or *bla*_IMP-1_ gene_,_ and 29 non-CPE isolates. The 66 CPE were characterised using either the Resist-4 O.K.N.V. kit (Coris BioConcept, Gembloux, Belgium) or the LightMix^®^ multiplex polymerase chain reaction (PCR) carbapenemase kit (TibMolBioL, Berlin, Germany) and the results were used as the reference genotypes. In addition, 10 uncharacterised non-fermenter Gram-negative bacteria were included in the study to determine the performance of the assay in detecting carbapenemases in these organisms.

### Xpert Carba-R assay performance

To determine the accuracy, sensitivity, and specificity of the Xpert Carba-R assay, the 95 bacterial cultures were processed according to the manufacturer’s instructions. Briefly, bacterial suspensions corresponding to the 0.5 McFarland turbidity standard (Thermo Fisher, Johannesburg, South Africa) were prepared from fresh cultures. Thereafter, 10 mL of each suspension was transferred into a sample reagent vial, vortexed for 10 s, and 1.7 mL of the sample mixture was transferred into the sample chamber of the Xpert cartridge for processing.

Due to challenges in obtaining clinical rectal swab specimens positive for CPE in the absence of an outbreak, rectal swabs spiked with the 95 characterised isolates were used. First, stool samples provided voluntarily by researchers involved in this study were screened for the presence of carbapenemases using the Xpert Carba-R assay as it can be performed directly from the stool and does not require culture. Thereafter, for each of the 95 isolates, 10 mL of a 0.5 McFarland bacterial suspension was added to 990 mL of the negative stool matrix and the mixture was seeded onto the sterile rectal swabs as per Cepheid’s acceptance swab criteria, and then processed following the manufacturer’s instructions.

A true positive was defined as when the expected carbapenemase genes were detected by the assay and a true negative was defined as when no carbapenemase gene was detected in non-CPE isolates and the CPE-negative rectal swabs. A discordant result was defined as an Xpert Carba-R assay result that was different from the reference genotype. Isolates with discordant results were sent to the National Institute for Communicable Diseases for whole-genome sequencing (WGS) to confirm the presence or absence of the targeted carbapenemase genes.

For WGS, total genomic DNA was extracted using the QIAamp Mini Kit (Qiagen, Germantown, Maryland, United States). Sequencing was performed using the NextSeq Instrument (Illumina, San Diego, California, United States) with 2 × 150 base pairs paired-end sequencing per flow cell and the paired-end reads were filtered for quality (Q > 30 and length > 50 base pairs) using Trim Galore v0.6.2 (https://github.com/FelixKrueger/TrimGalore). *De novo* assembly was performed using SPAdes v3.13 (https://github.com/kbaseapps/kb_SPAdes). Acquired and chromosomal antimicrobial resistance genes were determined using ResFinder v4.1 (https://cge.cbs.dtu.dk/services/ResFinder) and the Comprehensive Antibiotic Resistance Database (https://card.mcmaster.ca/). Carbapenemases identified based on WGS data were considered as the final genotype for discordant isolates and used to determine the accuracy, sensitivity, and specificity of the assay. Data were captured on Microsoft Excel (Microsoft, Redmond, Washington, United States) and analysis was performed using the following equations:

$$\begin{array}{l} \text{Accuracy}=(\text{True negatives}+\text{True positives})/\\ (\text{True negatives}+\text{True positives}+\text{False negatives}+\\ \text{False Positives})\times 100 \end{array}$$ [Eqn 1]

$$\begin{array}{l} \text{Sensitivity}=(\text{True positives})/\\ (\text{True positives}+\text{False negatives})\times 100 \end{array}$$ [Eqn 2]

$$\begin{array}{l} \text{Specificity}=(\text{True negatives})/\\ (\text{True negative}+\text{False positives})\times 100 \end{array}$$ [Eqn 3]

To determine sample stability, spiked rectal swabs were stored at room temperature and tested using the Xpert Carba-R assay on different days: day 0 (day of preparation), 1, 3, 5, and 7. The limit of detection of the assay was also determined by preparing serial dilutions up to 1.5 × 10^1^ colony forming units (CFU)/mL from 0.5 McFarland suspensions of CPE positive for *bla*_KPC_, *bla*_NDM_, *bla*_VIM_, *bla*_OXA-48_, and *bla*_IMP-1_. Each dilution was tested using the Xpert Carba-R assay. Lastly, the performance of the Xpert Carba-R assay was tested on non-fermenter organisms. Carbapenemases detected in non-fermenter organisms were confirmed using the Resist-4 O.K.N.V. kit (Coris BioConcept, Gembloux, Belgium) and were subjected to WGS at the National Institute for Communicable Diseases in Johannesburg, South Africa. These results were not included in the accuracy, sensitivity, and specificity calculations for the assay.

## Results

The Xpert Carba-R assay correctly identified all of the carbapenemases in 94% (61/66) of the tested isolates with a cycle threshold ranging between 20.6 and 27 for culture and between 24.5 and 30.6 for spiked rectal swabs. The assay further detected all double carbapenemase-producing isolates (*bla*_NDM_+*bla*_OXA-48_) and reported all 29 non-CPE isolates as negative for carbapenemases (Table 1; online Supplementary Table 1).

**TABLE 1:** Performance of Xpert Carba-R assay for the detection of carbapenemases in cultured bacterial isolates and spiked rectal swab specimens at the Infection Control Laboratory Services in Johannesburg, South Africa, April 2021 – September 2021.

Organism	Reference genotype (*n* = 95)	Xpert Carba-R C+R	Sensitivity (%)	Specificity (%)
***bla*_VIM_ (*n* = 10)**				
*Klebsiella pneumoniae*	5	TP	100	100
*Enterobacter cloacae*	2	TP		
*Klebsiella variicola*	1	TP		
*Klebsiella oxytoca*	2	TP		
***bla*_NDM_ (*n* = 15)**				
*Klebsiella oxytoca*	1	TP	100	100
*Proteus* species	1	TP		
*Enterobacter cloacae*	3	TP		
*Escherichia coli*	1	TP		
*Klebsiella pneumoniae*	6	TP		
*Serratia marcescens*	1	TP		
*Citrobacter freundii*	1	TP		
*Enterobacter* species	1	TP		
***bla*_NDM+OXA-48_ (*n* = 5)**				
*Klebsiella pneumoniae*	3	TP	100	100
*Enterobacter cloacae*	2	TP		
***bla*_KPC_ (*n* = 13)**				
*Enterobacter cloacae*	4	TP	89	100
*Klebsiella pneumoniae*	1	TP		
*Citrobacter freundii*	2	TP		
*Serratia marcescens*	1	TP		
*Klebsiella pneumoniae*	5	Discordant†		
***bla*_OXA-48_ (*n* = 20)**				
*Klebsiella pneumoniae*	15	TP	100	97
*Serratia marcescens*	1	TP		
*Enterobacter cloacae*	3	TP		
*Escherichia coli*	1	TP		
***bla*_IMP_ (*n* = 3)**				
*Escherichia coli*	1	TP	100	100
*Acinetobacter baumannii*	1	TP		
*Pseudomonas aeruginosa*	1	TP		
**Non-CPE (*n* = 29)**				
*Klebsiella pneumoniae*	14	TN	N/A	N/A
*Enterobacter cloacae*	3	TN		
*Escherichia coli*	9	TN		
*Enterobacter asburiae*	1	TN		
*Klebsiella oxytoca*	1	TN		
*Enterobacter* species	1	TN		

Note: Reference genotypes were previously determined using Resist-4 O.K.N.V or LightMix^®^ multiplex PCR carbapenemase kit.

C+R, results for both cultured isolates and spiked rectal swabs; TP, true positive; TN, true negative; N/A, not applicable; CPE, carbapenemase-producing *Enterobacterales*.

† , Discordant results were resolved with whole-genome sequencing.

Five isolates previously characterised as KPC-producing *K. pneumoniae* using the LightMix^®^ multiplex PCR carbapenemase kit were negative for the *bla*_KPC_ gene on the Xpert Carba-R assay as well as on Resist-4 O.K.N.V. Three of these isolates were positive for the *bla*_NDM_ gene while the other two isolates were positive for the *bla*_VIM_ (isolate 42) and *bla*_OXA-4__8_ (isolate 43) genes.

Whole-genome sequencing confirmed the presence of *bla*_NDM-1_ and the absence of the *bla*_KPC_ in the three *K. pneumoniae* isolates; these isolates were thus true positives for *bla*_NDM_. Whole-genome sequencing results also showed that isolate 42 was negative for the *bla*_KPC_ gene, and harbored *bla*_VIM-1_, which corroborated the results of the Xpert Carba-R assay. This isolate was considered a true positive for *bla*_VIM_. Further, WGS detected the presence of the *bla*_KPC-2_ gene in isolate 43, which was not detected by the Xpert Carba-R assay and Resist-4 O.K.N.V kit. Interestingly, the Xpert Carba-R assay and Resist-4 O.K.N.V kit both detected the presence of the *bla*_OXA-48_ gene, which was not detected by WGS. Isolate 43 was, therefore, considered a false negative for *bla*_KPC_ and a false positive for *bla*_OXA-48_ (Table 2).

**TABLE 2:** Investigation of five discordant results obtained using the Xpert Carba-R assay at the National Institute for Communicable Diseases, Johannesburg, South Africa, April 2021 – September 2021.

Isolate number	Reference genotype	Resist-4 O.K.N.V	Xpert Carba-R C+R	WGS	Outcome
39	*bla*_KPC_	*bla*_NDM_	*bla*_NDM_	*bla*_NDM-1_	TP for *bla*_NDM_
40	*bla*_KPC_	*bla*_NDM_	*bla*_NDM_	*bla*_NDM-1_	TP for *bla*_NDM_
41	*bla*_KPC_	*bla*_NDM_	*bla*_NDM_	*bla*_NDM-1_	TP for *bla*_NDM_
42	*bla*_KPC_	*bla*_VIM_	*bla*_VIM_	*bla*_VIM-1_	TP for *bla*_VIM_
43†	*bla*_KPC_	*bla*_OXA-48_	*bla*_OXA-48_	*bla*_KPC-2_	FN for *bla*_KPC;_ FP for *bla*_OXA-48_

Note: The isolates were re-tested using the Resist-4 O.K.N.V kit and subjected to whole-genome sequencing.

WGS, whole-genome sequencing; C+R, results for both cultured isolates and spiked rectal swabs; Outcome, results of the Xpert Carba-R assay as compared to WGS as the gold standard; TP, true positive; FP, false positive; FN, false negative.

† , Isolate number 43 was false negative for *bla*_KPC_ and false positive for *bla*_OXA-48_.

The overall accuracy of the Xpert Carba-R assay was 98%, with a sensitivity of 98% and specificity of 97%. For individual gene targets, the sensitivity and specificity were 100% for *bla*_NDM_, *bla*_VIM_, and *bla*_IMP-1_. The sensitivity for *bla*_KPC_ was 89% while specificity was 100%, whereas for *bla*_OXA-48_, the sensitivity was 100% and the specificity was 97%. The limit of detection for the Xpert Carba-R assay was 1 × 10^1^ CFU/mL for the *bla*_KPC,_ *bla*_VIM__,_ and *bla*_NDM_ genes and 1 × 10^2^ CFU/mL for the *bla*_OXA-48_ and *bla*_IMP_ genes. The spiked rectal swabs were still positive on day 7 when stored at room temperature and the assay was found to be 100% reproducible.

The assay was also able to detect carbapenemase genes in non-fermenting Gram-negative bacteria. Two *Acinetobacter baumannii* isolates were positive for *bla*_NDM_ and one *Pseudomonas aeruginosa* isolate was positive for *bla*_VIM_. Whole-genome sequencing confirmed the presence of the *bla*_NDM_ and *bla*_VIM_ genes in these non-fermenter organisms (Table 3).

**TABLE 3:** Performance of Xpert Carba-R assay for the detection of carbapenemases in 10 non-fermenter organisms compared to the Resist-4 O.K.N.V kit and whole-genome sequencing performed at the National Institute for Communicable Diseases, Johannesburg, South Africa, April 2021 – September 2021.

Organism	Resist-4 O.K.N.V	Xpert Carba-R	WGS
*Acinetobacter baumannii*	*bla*_NDM_	*bla*_NDM_	*bla*_NDM-1_, *bla*_OXA-23_, *bla*_OXA-69_
*Acinetobacter baumannii*	Neg	Neg	N/A
*Pseudomonas aeruginosa*	Neg	Neg	N/A
*Pseudomonas aeruginosa*	*bla*_VIM_	*bla*_VIM_	*bla*_VIM-2_, *bla*_OXA-846_, *bla*_OXA-395_
*Pseudomonas aeruginosa*	Neg	Neg	N/A
*Stenotrophomonas maltophilia*	Neg	Neg	N/A

WGS, whole-genome sequencing; N/A, not applicable – these isolates were not sent for WGS; Neg, negative for the five common carbapenemases.

## Discussion

In this evaluation study, the Xpert Carba-R assay demonstrated high performance with 98% sensitivity and 97% specificity when tested on cultured isolates on blood agar as well as on spiked rectal swabs. The results from this study are in agreement with the manufacturer’s information and corroborate results from other evaluation studies conducted in Europe and the United States which demonstrated high sensitivity and specificity.^11,12,13^ The assay was able to detect double carbapenemases (*bla*_NDM_ and *bla*_OXA-48_) in three *K. pneumoniae* and two *Enterobacter cloacae* isolates. The occurrence of two or more carbapenemase resistance genes in CPE is not an unusual phenomenon and has been previously reported in strains of *K. pneumoniae* and *Escherichia coli* isolated from Polish tourists surviving the terrorist attack at the Bardo Museum in Tunisia.^14^ More recently, the occurrence of *bla*_NDM_ and *bla*_OXA-48_ carbapenemases was reported in an outbreak caused by *K. pneumoniae* in Germany and in *K. pneumoniae* strains in Greece.^15,16^

The Xpert Carba-R assay was also able to detect carbapenemases in three non-fermenters. This is in agreement with the manufacturer’s claim that the Xpert Carba-R assay can be performed on pure colonies of *Enterobacterales, Acinetobacter baumannii, or Pseudomonas aeruginosa*. It is worth noting that the Xpert Carba-R assay has an intrinsic limitation in that it cannot detect carbapenemases that are not part of its test repertoire such as those detected by WGS in this study, including *bla*_OXA-23_, which is a potent carbapenemase that spreads via plasmid-mediated transfer and is predominantly found in clinical *A. baumannii* isolates.^17^ Although routine screening is not currently recommended for non-fermenter organisms, their increasing prevalence raises questions of concern.^18^ Further studies are necessary to understand the extent of carbapenemases in non-fermenter organisms and their role in the spread of these enzymes in healthcare settings.

The limit of detection claims by Cepheid for detecting the carbapenemases in rectal swabs ranges between 1 × 10^1^ CFU/swab and 1 × 10^2^ CFU/swab. Furthermore, Cepheid recommends that rectal swabs collected using the rectal specimen collection device (part number 900-0370) can be stored at room temperature for up to five days. These findings were corroborated by our verification. Additionally, spiked rectal swabs remained positive for up to seven days when stored at room temperature.

### Limitations

This study had a few limitations. First, the performance of the assay on rectal specimens was tested using spiked rectal swabs instead of clinical specimens. Second, the limit of detection was only tested on cultured isolates and not spiked rectal swabs. Lastly, due to the low prevalence of IMP-producing *Enterobacterales*, only three isolates were used in the verification, and these included two non-fermenter organisms.

### Conclusion

We concur with other studies that the Xpert Carba-R assay offers a reliable and fast method for the detection of the five most common carbapenemases in carbapenemase-producing organisms. The assay can be performed on rectal swab specimens or pure bacterial cultures of carbapenem-nonsusceptible isolates and provides automated results in less than an hour. The assay can detect double carbapenemase producers as well as carbapenemases in non-fermenter organisms. The Xpert Carba-R assay can, therefore, aid in the timeous and appropriate institution of treatment and infection prevention and control measures.
